# Optimizing the Routine Use of Clinical Guidelines by Addition of Supplements (Probiotics and/or Bismuth) to *Helicobacter pylori* Eradication Protocols in a Clarithromycin Resistant and Tetracycline/Bismuth Naive Area: A Real-World Data Retrospective Analysis of 402 Cases (2016–24) in a Single Gastroenterology Unit

**DOI:** 10.3390/antibiotics14090870

**Published:** 2025-08-29

**Authors:** András Gelley, Noémi Kéri, Péter Birinyi, Kinga Komka, Vajk Hardy, László Döngölő, Dóra Szeli, Ibolya Czegle

**Affiliations:** 1 Department of Internal Medicine, Gastroenterology and Endocrinology, Buda Hospital of the Hospitaller Order of Saint John of God, H-1027 Budapest, Hungary; gelley.andras@irgalmas.hu (A.G.); hardy.vajk@irgalmas.hu (V.H.); dongolo.laszlo@irgalmas.hu (L.D.); szeli.dora@irgalmas.hu (D.S.); 2 Department of Intensive Therapy, Faculty of Medicine, Semmelweis University, H-1085 Budapest, Hungary; keri.noemi@semmelweis.hu; 3 Department of Pharmacodynamics, Faculty of Pharmacy, Semmelweis University, H-1085 Budapest, Hungary; birinyi.peter@semmelweis.hu; 4 Department of Chemical and Environmental Process Engineering, Faculty of Chemical Technology and Biotechnology, Budapest University of Technology and Economics, H-1111 Budapest, Hungary; komka.kinga@vbk.bme.hu; 5 Department of Internal Medicine and Hematology, Faculty of Medicine, Semmelweis University, H-1085 Budapest, Hungary

**Keywords:** Helicobacter pylori, clinical guideline, eradication protocol, bismuth, probiotics

## Abstract

**Background:** The official current guideline for *Helicobacter pylori* (*H. pylori*) eradication is to use tetracycline–bismuth-based protocols as first line treatment due to the increasing incidence of clarithromycin resistance in the last decade. The unavailability of tetracycline and bismuth-containing medicines, however, is an issue in many countries, limiting the routine use of these protocols. The value of using additional probiotics in eradication protocols is also unclear. Direct comparison data on the effect of available bismuth compounds and different probiotic strains on eradication outcome are limited. **Goal:** The aim of our investigation was to find optimal eradication protocols, supplementations and treatment duration for routine clinical use in our gastroenterology unit, located in a highly clarithromycin-resistant and tetracycline–bismuth-naïve area. **Materials and Methods:** We conducted a retrospective real-world data analysis of 402 *H. pylori* positive patients between 2016 and 2024. *H. pylori* infection was diagnosed using histological examination of gastroscopy samples obtained from the gastric antrum. For the evaluation of treatment success or failure, ^14^C breath tests and stool *H. pylori* antigen tests were performed. Data on patient characteristics and treatment protocols were collected from our electronic patient record system, and treatment success was compared between the different treatment regimes. **Results:** Despite the regional clarithromycin resistance, supplementing clarithromycin-based regimens with bismuth and probiotic during the 14-day treatment duration showed a high and comparable cure rate when compared to tetracycline-based regimens, which are the current first-line therapies. When tetracycline-based combination is available, it is recommended to use it with an additional probiotic to achieve the best possible outcome. Comparison of the effect of available bismuth preparations on treatment success showed no significant difference. Generally, probiotic-containing protocols are more successful, compared to those treatments without this supplement. There was no statistical difference in the cure rates amongst the four probiotic strains used, where sample size allowed statistical analysis. Furthermore, supplementation with probiotics *Lactobacillus reuteri ATCC PTA 6475* or *Lactobacillus reuteri Protectis^®^ DSM 17938* showed promising high treatment success rates (85.2% and 100.0%, respectively) in our study.

## 1. Introduction

*H. pylori* is still one of the most common human bacterial infections worldwide. Its eradication remains a critical public health goal due to its negative effects, such as its role in causing infection-associated cancer.

Current consensus guidelines (the American College of Gastroenterology (ACG) 2024 Guideline and Maastricht VI/Florence consensus) recommends susceptibility-guided or local antibiotic resistance-guided treatments instead of empirical eradication regimens. Our short guideline overview is focused only on the two most popular clarithromycin-based triple and tetracycline-based quadruple regimens. In regions, where primary clarithromycin resistance exceeds approximately 15%, and in cases where no previous antibiotic susceptibility testing is available, a 14-day tetracycline-based quadruple therapy (consisting of a PPI, bismuth, metronidazole, and tetracycline) is advised generally as first line treatment. In our area, the empirical use of the classical clarithromycin-based triple therapy (PPI + clarithromycin + amoxicillin or metronidazole) is not recommended as first line therapy. In regions, where estimated clarithromycin resistance is under 15%, both the amoxicillin–clarithromycin-based triple therapy (amoxicillin + clarithromycin + PPI) and the above-mentioned tetracycline-based quadruple therapies are recommended as possible first line treatments. Furthermore, in countries where commercial preparations containing antibiotics and bismuth are available, these can be given with PPI to achieve better patient compliance, with success rates exceeding 90%. Eradication rates using PPI–clarithromycin–amoxicillin/metronidazole triple therapies decreased over time, due to the increasing prevalence of clarithromycin resistance related to the frequent use of macrolide antibiotics in clinical practice [1,2].

A wide range—from 5.5% (macrolide-naïve patients) to 17.3%of estimated *H. pylori* clarithromycin resistance is reported in Hungary, based on the literature [3,4]. In routine clinical practice, therefore, no equivocal data are available to guide treatment decisions. Hetero-resistance—where resistant and susceptible strains coexist however, may occur in ~7% of cases, which contributes to treatment failure even below classical thresholds [5]. Based on the literature, and the continuous increase in the use of macrolide antibiotics, Hungary is considered as a clarithromycin-resistant area. Empirical eradication therapy in Hungary has typically used the amoxicillin–clarithromycin-based triple therapy in treatment-naïve patients. With rising macrolide use over time, however, treatment failures have become more common [6]. Recent trends in European practice, using the Hp-EuReg registry, emphasize a shift toward a single capsule bismuth quadruple therapy, achieving eradication rates of 90–95% both as first line and rescue therapy in resistant settings. These results can only be achieved by a minimum of 10 days of eradication treatments [7]. Although there is limited Hungary-specific data, the overall Hungarian antibiotic resistance pattern likely mirrors the broader Central European pattern, resulting in a general treatment success rate of around 80% [8]. Based on published data, *H. pylori* treatment success rate in Hungary is comparable with the international rate when tetracycline-based quadruple therapies were used (93.6%). Conventional clarithromycin-based regimens, however, only had a much lower, 75%, eradication rate [9].

Regarding pharmaceutical access, the availability of tetracycline and bismuth salts (as individual agents and in fixed dose combinations) are limited in Hungary. Tetracycline is only available by individualized drug import; and bismuth drugs are only available in the form of bismuth oxide. Bismuth citrate, which is pharmacologically similar to the commercially available bismuth oxide, is available as a magistral preparation. A combination drug approved in Europe since 2009 that contains bismuth sub-citrate, metronidazole and tetracycline is not yet available in Hungary. Tetracycline and bismuth compounds are available as prescription medications in Hungary, but they are historically under-used due to entrenched triple therapy prescription habits. Furthermore, the Hungarian National Formulary gives the opportunity to prepare magistral bismuth–metronidazole, but it has a very limited availability (it is prepared only in one pharmacy) for use in routine clinical settings.

Hungary can therefore generally be considered a tetracycline and bismuth naïve area, so a possible shift towards using tetracycline containing eradication regimens could be a promising direction to make *H. pylori* eradication rates comparable to international statistics. As previously mentioned, despite the limitation of its availability, the classic bismuth-containing quadruple therapy (PPI, bismuth, tetracycline, and metronidazole) has been recommended as a first-line therapy in areas with high and uncleared clarithromycin resistance.

Tetracycline-based regimens typically contain bismuth as a supplement. It is well known that addition of bismuth to a 14-day standard triple therapy with clarithromycin and amoxicillin eradicates *H. pylori* infection in more than 90% of patients, resulting in a potential therapeutic gain (10–20%) in populations with moderate to high clarithromycin resistance, with an acceptable safety profile and adherence rate [8]. Although a range of data support bismuth addition to any eradication protocols [10,11,12], comparison of the different currently available bismuth compounds (commercial bismuth oxide, magistral bismuth–citrate and bismuth–metronidazole) have not yet been compared for eradication efficiency. Meta-analyses and clinical studies also support the addition of probiotics to standard treatment protocols, showing that these adjuvant probiotic supplements modestly increase eradication rates while reducing antibiotic related side effects, especially when combined with tetracycline-based quadruple therapy [13]. Data from the European registry also indicate that more frequent probiotic co-prescription is associated with improved tolerability and possibly better treatment adherence [14,15].

Limited data are available on the effect of different probiotic strains in this setting, with no data on the direct comparison of their effect on treatment efficacy. In many original articles, reviews, and meta-analyses, assessment of clinical symptoms was the trial outcome, with a presumed positive eradication result. In contrast, in this study, we reviewed treatment success rates without collecting patient-reported outcomes. A large meta-analysis of 25 randomized controlled trials (RCTs-3769 patients) evaluating single strains as adjuncts to standard eradication therapy found that only *Saccharomyces boulardii* CNCM I-745 significantly improved eradication rates. *Lactobacillus rhamnosus GG*, *Lactobacillus acidophilus*, *Lactobacillus casei*, *Lactobacillus reuteri*, *Clostridium butyricum* showed no clear benefit on eradication success [13]. Another high-quality systematic review (2025; 5036 cases, 19 RCTs) confirmed that the addition of *Saccharomyces boulardii* significantly increased eradication rates [16]. The efficacy of Lactobacillus strains was investigated by a comprehensive RCT involving *Lactobacillus reuteri DSM17648* as adjunct to standard therapy, achieving eradication rates exceeding 90% [17]. However, pooled RCTs in meta-analyses failed to show a statistically significant improvement in eradication rates for *Lactobacillus reuteri*. As a head-to-head or combined probiotic comparison, a smaller RCT combined *Saccharomyces boulardii* and *Lactobacillus reuteri* with quadruple therapy. While data on direct comparison was limited, the combined regimen suggested a higher eradication rate and better tolerability. The sample size, however, was small, and no definitive strain efficacy separation was provided [18].

Besides the lack of data for direct comparison of probiotic strains, their unique additional effect on different treatment protocols is still an open question. Combination use of both supplements (bismuth and probiotics) has not yet been examined regarding efficacy; however, promising potential can be foreseen in further enhancement of eradication success.

The aim of our retrospective real world data analysis of 402 cases was to give a comprehensive view and an optimalization of the routine use of clinical guidelines in *H. pylori* eradication in a clarithromycin-resistant and tetracycline-bismuth-naive area by covering the following topics: (1) description of trends regarding the number of eradication cases along with the protocols used, their treatment lengths and success rates, and the frequency of supplements used throughout the years in our clinic; (2) investigation of overall treatment protocol success rates; (3) determining the optimal duration of treatment; (4) finding the treatment protocol(s) that achieve the highest eradication rates; (5) clarifying whether the addition of probiotics improves treatment outcomes in different settings; (6) clarifying the effect of adding bismuth to clarithromycin-based protocols, and investigating the possible difference between bismuth compounds regarding treatment success; (7) finding the most effective probiotic strain and determining future efforts to improve patient care.

## 2. Results

### 2.1. Patient and Study Characteristics

**Figure 1:**
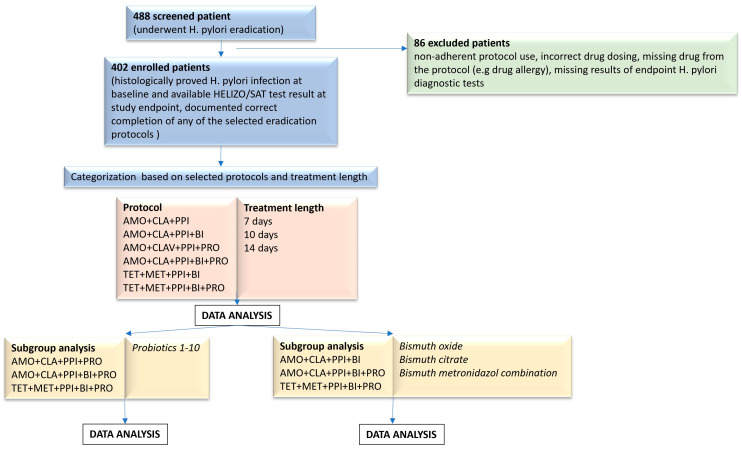
Study flowchart.

A total of 488 case files were screened initially of patients who underwent *H. pylori* eradication. A total of 402 patients met the inclusion criteria and were included in our analysis. A total of 86 cases were excluded due to non-adherence to treatment, incorrect drug dosing, drug removed from the treatment protocol (e.g., due to drug allergy), or missing results of endpoint *H. pylori* diagnostic tests (see Table 1). After enrollment, patient data were categorized according to treatment protocol, treatment length, and supplementation. Subgroup analyses were carried out in probiotic-containing cases to investigate the additional effect of different probiotic strains and in bismuth-containing cases to compare the effectivity of different bismuth compounds. (Figure 1, Study flowchart).

### 2.2. Trends in H. Pylori Eradication Between 2016 and 2024

**Figure 2:**
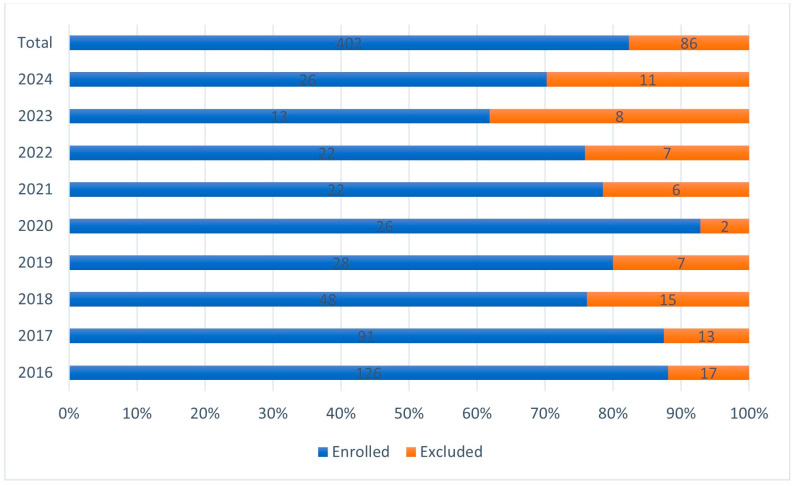
Proportion of enrolled and excluded cases.

**Figure 3:**
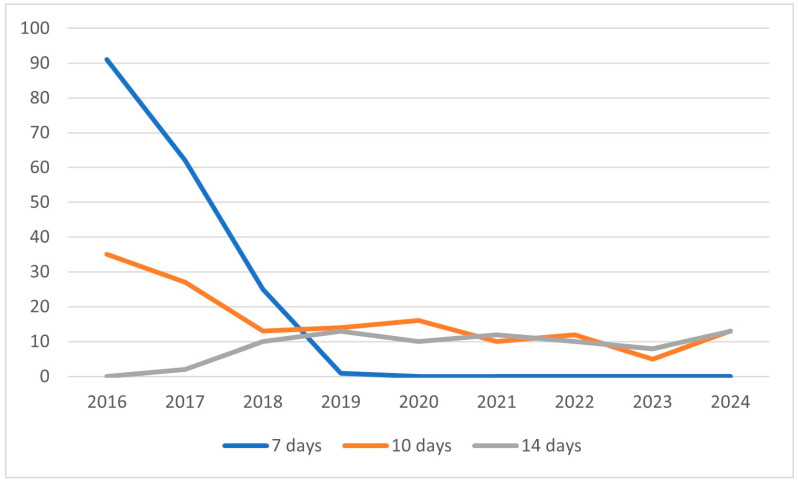
Trends in duration of eradication protocols.

**Figure 4:**
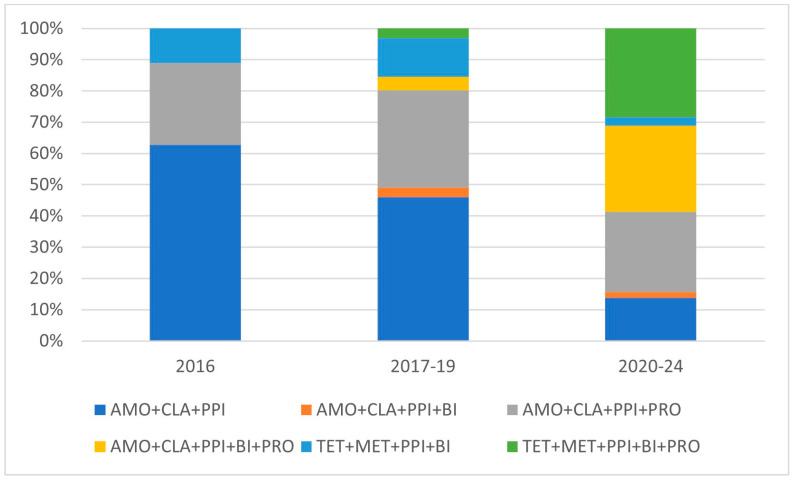
Proportion of different eradication protocols in study observational period.

Although a continuous and sharp yearly decline was observed in the number of eradication cases, the proportion of excluded cases and success rates remained the same (Figure 2). The incidence of clarithromycin-based protocols showed a continuous decline, whereas tetracycline-based regimens increased. A continuous decrease in the incidence of 7-day treatment protocols was seen that reached zero in 2019, with the proportion of 10- and 14-day protocols remaining the same after 2018 (Figure 3). Despite local clarithromycin resistance rates, the most popular protocol was the basic AMO + CLA + PPI, although a continuous decline in yearly case numbers was observed. The proportion of tetracycline-based protocols, as well as supplemented AMO protocols, continuously increased through our observational period. The use of probiotic-containing protocols became more and more popular, and by the end of the study period, in 2023–2024, all eradication regimens included probiotic supplementation (Figure 4). In Figure 4, the division of the 10-year study period was based on the proportion of probiotic-containing cases to appropriately show their continuous increase from year to year.

### 2.3. Investigation of Protocol Success Rates, Optimal Treatment Length, Determining the Best Eradication Protocol

Two basic protocols were supplemented with bismuth and/or probiotics; therefore, the overall success rate of six different treatment types were examined. As seen in Table 2, the most promising protocol was TET + MET + PPI + BI + PRO regarding success rates, followed by the two other clarithromycin-based protocols with supplements (AMO + CLA + PPI + PRO, AMO + CLA + PPI + BI + PRO). Statistical analysis based on the used antibiotic combination types did not find any difference in overall success rates (*p* = 0.5902, clarithromycin-based protocols vs. tetracycline-based regimens) (Table 3). Generally, probiotic-containing protocols were more successful (*p* = 0.0295) compared to those that did not contain this supplement (Table 4). The efficacy of therapy protocols and their treatment lengths were also investigated: for determination of optimal treatment duration, overall success rates of 7-, 10- and 14-day protocols were compared, showing significantly higher (*p* = 0.0056) treatment success rates with the 14-day protocols when compared to the 7- and 10-day treatment lengths (Table 2). Further subgroup analysis was performed to investigate eradication success rates amongst the 14-day probiotic-containing protocols only. Therapy success rate significantly increased using probiotic-containing 14-day treatment protocols (*p* = 0.0002) when compared to the non-probiotic-containing regimens in the same 14-day setting (Table 4). Interestingly, AMO + CLA + PPI + PRO (90.0%), AMO + CLAV + PPI + BI + PRO (95.0%), and TET + MET + PPI + BI + PRO (100.0%) have comparable treatment outcome results (Table 2).

### 2.4. Investigation of Additional Probiotic and/or Bismuth Effect on Success Rates

To further investigate the general positive effect of probiotics on treatment outcome, we examined separately its effect on AMO and TET protocols. Based on the known ameliorating effect of bismuth supplementation in antibiotic treatments, we investigated the possible additional effect of bismuth supplementation for the two different regimens.

#### 2.4.1. AMO Protocols

The success rate of clarithromycin-based–probiotic-containing protocol (AMO + CLA + PPI + PRO) and its bismuth supplemented version (AMO + CLA + PPI + BI + PRO) vs. the clarithromycin-based basic regimen (AMO + CLA + PPI) was examined: the addition of probiotics failed to show a significant difference in treatment success for any of the treatment durations (7, 10 and 14 days, *p*-values are in Table 5), although success rates show a marked increase in a 14-day setting (83 vs. 95%) when both supplement compounds were used. Despite the promising success rates in a 14-day setting, a possible cause of our non-significant result is the low case number in this sub-category (Table 5). Addition of bismuth itself does not cause any marked elevation of treatment success in clarithromycin-based combinations (AMO + CLA + PPI + BI vs. AMO + CLA + PPI) (Table 2)

#### 2.4.2. TET Protocols

In TET regimens, addition of probiotics to the eradication protocol resulted in a significant increase in success rate in the 14-day treatment setting (*p* < 0.0001). Similarly to AMO protocols, however, this effect was not significant with 10-day treatment durations. No data were available for the 7-day treatment protocols for investigation of a probiotic effect (Table 6).

Generally, comparison of all probiotic-containing protocols (186 cases) vs. all non-probiotic regimens (216 cases) showed significant increase in success rates (Table 4). In subgroup analysis, however, probiotic use was only significant in TET protocols, using 14-day treatment length.

### 2.5. Bismuth and Bismuth + Probiotic Combination Effect on Success Rates

Investigation the effect of bismuth itself was challenging in our study. In AMO protocols, the addition of bismuth had no significant effect on treatment success rates. TET protocols, which always contained bismuth (TET + MET + PPI + BI), could, however, be used for analysis, where we compared the effect of all bismuth-containing protocols (AMO + CLA + PPI + BI and TET + MET + PPI + BI vs. AMO + CLA + PPI) (Table 7). Our analysis, however, did not show any significant difference amongst these groups. The addition of probiotics to all bismuth-containing regimens (comparison of AMO + CLA + PPI + BI + PRO and TET + MET + PPI + BI + PRO vs. AMO + CLA + PPI + BI and TET + MET + PPI + BI), however, showed a significant increase in treatment success. This phenomenon was observed both when we made the comparison using all the cases (*p* = 0.0124) and when we only analyzed the 14-day treatment regimens (*p* < 0.0001). We can therefore say that additional probiotics significantly increase the efficacy of bismuth-containing treatment protocols in our cohort (Table 8).

### 2.6. Investigation of Bismuth Compounds

In our database search, regimens containing different bismuth preparations (commercially available bismuth oxide, magistral prepared bismuth citrate and a magistral combination of bismuth and metronidazole) were collected for further analysis. Comparing the effect of the three preparations used, the highest treatment success rate was observed when using a commercial bismuth oxide and magistral bismuth–metronidazole combination (90.3%), with no significant effect of type of bismuth choice (*p* = 0.0670) on success rates (Table 9).

### 2.7. Additional Value of Probiotic Choice by Investigating Different Preparations (Probiotics 1–10)

As previously described, there is a positive effect of probiotic use in our study with many treatment protocols. We therefore have further investigated this phenomenon by comparing the effect of ten different probiotic preparations used during the observational period. Probiotic compounds were anonymized for analysis by using serial numbers in the order of occurrence in our data search (see below in Table 10). Statistical analysis was only performed when the sample size was large enough to allow that. With AMO + CLA + PPI + PRO treatment protocols (a total of 82 cases), no statistical difference (*p* = 0.8380) was found between the use of Probiotic 1, Probiotic 2, Probiotic 3 or Probiotic 5 preparations (Table 11). Regarding the AMO + CLA + PPI + BI + PRO treatment protocol, 33 of the total 37 cases were supplemented with Probiotic 8 (27 cases) and Probiotic 6 (6 cases), with a success rate of 85.2% (23/27 cases) and 100% (6/6), respectively, and with no significant difference between the groups (*p* = 0.3146). With the TET + MET + PPI + BI + PRO treatment protocols, 27 out of 33 cases were supplemented with Probiotic 8, showing a success rate of 100% (27/27), with only 6 cases in this group with supplementation using different strains. Statistical analysis, therefore, could not be performed. Subgroup analysis treatments comparing probiotic treatments to “single strain” and “multiple strain” preparations also did not show any statistical difference (*p* = 0.2042) regarding success rates (Table 11).

It is, however, interesting that 27 patients receiving AMO + CLA + PPI + BI + PRO, and 27 further patients receiving TET + MET + PPI + BI + PRO treatments in our study receiving probiotic supplementation using Probiotic 8 had a treatment success rate of 85.2 and 100%, respectively. No further statistical analysis was possible, due to low sample size, using other probiotic strains in our study. This observation of 54 cases requires, therefore, further investigation in a prospective randomized trial or in a retrospective real-world study, where individual probiotic strains could be compared with higher treatment numbers per group. Additionally, in 19 cases, probiotic type was not specified in the patient documentation in our study. Although the treatment success rate was high in these cases at 94.7% (18/19), data were not taken account in further analysis due to the lack of relevant information we could gain (Table 12).

## 3. Discussion

Our retrospective study is an internationally unique and comprehensive assessment of the success rate of different *H. pylori* eradication protocols that conducted in Hungary: 1. the amoxicillin–clarithromycin-based, and 2. the tetracycline-metronidazole-based protocols. Furthermore, the combination of three different treatment lengths, along with the use of two possible supplements (bismuth and probiotics), were compared in our study. Although previous studies are available describing gold standard one of these gold standard therapy protocols [19,20,21], or a modified version, along with other treatment protocols [22,23,24], comparison of the two current gold standard treatments along with the assessment of the usefulness of two promising supplements (bismuth and probiotics) was missing.

The first main result of this study is in line with previous publications and with the current treatment guidelines [7,8,25], showing the superiority of the 14-day treatment duration compared to the 7- and 10-day treatments. Our analysis showed that treatment success rates were significantly higher in the three probiotic-containing regimens investigated (AMO + CLA + PPI + PRO, AMO + CLA + PPI + BI + PRO, TET + MET + PPI + BI + PRO) when compared to no-probiotic-containing treatment protocols. Furthermore, our results proved that, despite high local clarithromycin resistance, comparable success rates could be achieved by supplementation of the amoxicillin–clarithromycin-based protocols with bismuth and probiotics. Another relevant result of our study is that the most prominent effect of probiotics on treatment success rates with both treatment protocols were observed when they were also supplemented with bismuth. Hungary being a tetracycline-bismuth naive area, it is not unexpected that the best treatment success rates were seen with tetracycline-based protocols. Although in international clinical guidelines this treatment combination is suggested as first line-treatment, due to issues around tetracycline`s availability in Hungary, amoxicillin–clarithromycin-based protocols were often used in our clinic, showing non-inferior treatment success rates with supplementation.

As previously mentioned in the introduction, earlier data are available on the effect of different bismuth compounds; however, results of their comparison on treatment success are missing. In this study, direct comparison of different bismuth compounds (e.g., bismuth oxide, bismuth citrate and bismuth–metronidazole) were analyzed, showing an equivalent treatment success rate amongst the different types of product. Based on our data, we can say that the addition of any type of bismuth supplementation to any 14-day treatment protocols that include probiotics showed better overall treatment success rates in our cohort.

In our study, further analysis was performed to compare the effect of these four probiotics where sample size allowed statistical analysis. Our results, however, showed no statistical difference in success rates amongst these groups. The analysis included Probiotic 1 (*Lactobacillus acidophilus*, *Lactobacillus bulgaricus*, *Bacillus coagulans*, *Bifidobacterium animalis lactis*, *Streptococcus thermophilus*), Probiotic 2 (*Enterococcus faecium L3*), Probiotic 3 (*Lactobacillus acidophilus* (*LA-5*), *Bifidobacterium* (*BB-12*), *Lactobacillus paracasei* (*L. CASEI 431*)) and Probiotic 5 (*Saccharomyces boulardii CNCM I-745*). In previous literature (see introduction section), only *Saccharomyces boulardii* was found to increase eradication success rates in RCTs, which also showed a beneficial effect in our study. Our data, however, also showed non-inferiority of the other species listed. Future randomized controlled clinical trials and retrospective studies should aim to confirm the usefulness of Lactobacillus and other strains in a bigger cohort.

An important finding of our study is that Probiotic 8 (Lactobacillus reuteri) supplementation, i.e., the “PRO” part of AMO + CLA + PPI + BI + PRO and TET + MET + PPI + BI + PRO treatment protocols, had a very high 85.2% and 100% treatment success rate, respectively. This finding also requires further studies with larger case numbers.

In conclusion, when following clinical recommendations is not possible due to limitations in tetracycline availability, comparable success rates can be achieved using clarithromycin-based protocols with probiotics and bismuth in a 14-day regimen. The cost of the two basic protocols is approximately the same, so cost considerations should not influence treatment choices in a real-world setting.

As this was a retrospective trial in which we collected real-world data on local clinical routines, a limitation existed in the statistical analysis and interpretation of results in that physician choice might have influenced the treatment protocol or the added probiotic strains. Therefore, as can be seen in Table 12, each treatment protocol was mainly supplemented by the same probiotics, as physicians used their preferred treatments for patient care, which caused a shift in case numbers, making statistical analysis impossible.

Besides clinical trials, several data sources from microbiological and other preclinical studies support the beneficial effects of probiotics on *H. pylori* eradication. As this study is a database search, only a brief explanation of the mechanism of action is provided here. Probiotics affect the colonization of *H. pylori* in several ways: they interfere with bacterial adhesion to epithelial cells [26]; inhibit bacterial enzyme activity (urease, catalase, and carbonic anhydrase); and interfere with the secretion of antibacterial substances (e.g., bacteriocins such as reuterin, lacticin, and bulgaricin) [27]. Probiotics also have a negative effect on biofilm formation and modulate the host cell immune response to infection by influencing the balance of pro- and anti-inflammatory cytokines (e.g., IL-8, IL-10, TNFα, IL-1β, and IL-4) [28,29,30]. They also regulate gastric microecology. Probiotic supplementation can regulate gastric flora structure and promote gastric microecological recovery, which is considered to be related to *H. pylori* eradication [31]. Especially, quadruple antibiotic therapy for *H. pylori* infection can exacerbate gastrointestinal microecological disorders. Probiotics, on the other hand, restore the balance of intestinal flora through different mechanisms, improve eradication rates, and reduce adverse reactions [32]. In conclusion, probiotics play an important role in the prevention and treatment of gastrointestinal diseases by antagonizing *H. pylori* colonization, regulating gastric pH, secreting antibacterial substances, stimulating immune responses, and regulating gastrointestinal flora [15,33,34].

Based on our data, a promising future direction would be to carry out a randomized clinical trial, inorder to directly compare the treatment success of the addition of probiotics to amoxicillin- vs. tetracycline-based bismuth regimens. A possible shift to longer (14-day) treatments raises safety and adherence issues that require further investigation. In addition, the optimal dosage and the choice of the best probiotic strain, along with treatment duration and timing of supplementation relative to antibiotics treatment, would also require further study and standardization before an official recommendation can be made (study results are summarized at Table 13).

## 4. Materials and Methods

### 4.1. Patients

In this real-world retrospective trial, the medical records of patients who underwent *H. pylori* eradication in our outpatient clinic were investigated in a nine-year period (2016–2024). A total number of 486 eradication cases were screened and 402 cases were selected who met the inclusion criteria and enrolled to our trial after exclusion of non-suitable cases (see exclusion criteria below) and underwent further data collection.

### 4.2. Diagnostic Tools

The initial diagnosis of *H. pylori* infection was performed on gastric antrum biopsies, using histological examination, which used Giemsa staining or immunohistochemistry (*H. pylori* rabbit monoclonal antibody manufactured by Sigma-Aldrich, St. Louis, MO, USA), the latter in cases where Giemsa staining showed questionable results. Envision Target Retrieval System was used for detection purposes. For the determination of treatment outcome, results of HELIZO™ exhalation tests (performed at the Nuclear Medicine Department of Buda Hospital of the Hospitaller Order of Saint John of God), or stool Helicobacter antigen (SAT) tests (provided by the Central Laboratory of Buda Hospital of the Hospitaller Order of Saint John of God) were used. The HELIZO™ exhalation tests are based on the ^14^C content of exhaled air, containing radioactively labelled carbamide. The HELIZO™ exhalation test and HELIPROBE™ equipment, used for detection, were manufactured by Isotope Institute Ltd, Budapest, Hungary. For stool antibody testing, DiaQuick *H. pylori* stool cassette (sensitivity 98.8%, specificity 98.4%, manufactured by DiaLab Ltd., Neudorf, Austria) were used.

### 4.3. Database Search and Case Screening

Data collection was performed trough institutional database search (EMMA™). The basis of patient screening in our study was a histologically proven *H. pylori* infection, along with the availability of either the HELIZO™ exhalation tests or stool Helicobacter antigen (SAT) tests at study endpoint.

### 4.4. Inclusion Criteria

We selected only patient data that met all the inclusion criteria: (1) the initial diagnosis of *H. pylori* infection, (2) the presence of the final result on eradication by the diagnostic tests (HELIZO™ or SAT) 6 to 8 weeks after completing eradication treatment, (3) and the availability of prescription details of antibiotics used for eradication (agent name or drug trade name and dose).

### 4.5. Exclusion Criteria 

Case documentations containing incomplete data (e.g., missing treatment length, exact type of medications), as well as treatments deviating from the protocol (e.g., alternative combinations due to the patient’s drug allergy, the patient’s intolerance to one of the components, or any conditions resulting in earlier termination of the prescribed treatment) were not included in the final statistical calculation. All eradication cases were recorded regardless of which therapy protocol (in first line, or after a failure as second or later attempt) the patient received.

### 4.6. Determination of Investigated Outcomes in Database

“Baseline- *H. pylori* infection” meant that the initial histology proved *H. pylori* infection. Cases where the result of HELIZO™ or SAT test were negative after completing eradication treatment were labelled “Success”. In contrast, positive HELIZO™ or SAT tests after treatment were labelled as “Failure”.

### 4.7. Antibiotics Combinations, PPI and Supplementations

Commercially available amoxicillin, clarithromycin and PPIs are most often used in our clinical practice. The tetracycline used was a commercially available medication that was available only via an individual import process through one pharmacy in Hungary. Several efforts were made to improve success rates in clinical routine by using bismuth compounds as a part of tetracycline-based regimes and as a supplement in clarithromycin-based protocols. Although the commercially available bismuth oxide is pharmacologically similar to the bismuth–citrate magistral preparation (in pharmacological nomenclature they are considered synonyms), due to their different preparation methods, they were treated as two separate entities in our study. In some cases, magistral bismuth–metronidazole capsules were also prescribed by a physician in our clinic. Regarding the probiotics, commercially available probiotic strains were used at the choice of the gastroenterologist.

### 4.8. Treatment Protocol

In line with the international recommendations, two standard types of eradication protocol, a clarithromycin-based (AMO + CLA + PPI), and an original tetracycline-based (TET + MET + PPI) were collected from the database. As Hungary is considered to be a clarithromycin-resistant area, and because the tetracycline and bismuth compound availability is limited, physicians altered the clarithromycin-based protocol by adding supplements, mainly probiotics, and in some cases bismuth compounds (AMO + CLA + PPI ± BI ± PRO) in order to improve treatment success rates. The tetracycline-based protocols normally contain bismuth, and in many cases they are supplemented with probiotics (TET + MET + BI ± PRO). Therefore, the following eradication therapies were seen in our clinic: (1) standard triple clarithromycin-based protocol containing amoxicillin, clarithromycin, proton pump inhibitor, (AMO + CLAV + PPI); (2) a quadruple clarithromycin-based combination containing amoxicillin, clarithromycin, proton pump inhibitor and bismuth (AMO + CLA + PPI + BIZ), (3) a quadruple clarithromycin-based protocol containing amoxicillin, clarithromycin, proton pump inhibitor with probiotics (AMO + CLA + PPI + PRO), (4) a quintuple protocol of amoxicillin, clarithromycin, proton pump inhibitor, supplemented both bismuth and probiotics (AMO + CLA + PPI + BIZ + PRO). The other types of investigated regimens were the standard (5) tetracycline + metronidazole + PPI + bismuth regimen (TET + MET + PPI + BIZ), (6) supplemented with probiotics (TET+ MET + PPI + BIZ + PRO) (Table 14 and Table 15). The treatment lengths of all the above combinations were 7, 10 or 14 days. The type of proton pump inhibitor was not examined in this study, therefore no details are provided. In all cases, however, PPIs were given in their standard prescription doses. Any other prescription or non-prescription medications were excluded from our data collection.

### 4.9. Investigation of Supplements

As a separate part of our study, we examined the additional value of ten different probiotic strains in those three protocols where they were used (AMO + CLA + PPI + PRO, AMO + CLA + PPI + BIZ + PRO, TET + MET + PPI + BIZ + PRO) (Table 15). Additionally, bismuth was given in three different formulations for these treatment protocols (AMO + CLA + PPI +BIZ, AMO + CLA + PPI + BIZ + PRO, TET + MET + PPI + BIZ, TET+ MET + PPI + BIZ + PRO), using appropriate dosing: (1) commercially available bismuth oxide, (2) magistral preparation of bismuth–citrate compound, (3) magistral preparation of bismuth–metronidazole combination (Table 16).

### 4.10. Database Setup

The data collected were saved in a Microsoft Excel table. Physicians were anonymized and marked with randomly assigned numbers. Collected data were as follows: date of birth and gender of the patient, length of the treatment, type of eradication protocol and medications used (antibiotics, bismuth and proton pump inhibitor and doses), additional probiotic or bismuth use (where applicable), and outcome of the therapy (success or failure) based on HELIZO™ or SAT test results. No data on patient reported outcomes were collected (e.g., symptoms, compliance) due to the retrospective nature of our study, and because the main goal of the study was to optimize social guidelines for our daily clinical practice.

### 4.11. Statistical Analysis

Statistical analysis was performed using TIBCO Statistica Software (version 14.1.0). The calculation was based on 2 × 2 or in some cases r × c contingence tables using the chi-square test. The significance level was 5%.

## Figures and Tables

**Table 1:** Patient characteristics.

Patients	Nr. (%)
Total	488
Enrolled	402 (82.4)
Excluded	86 (17.6)
Success	311 (77.3)
Failure	91
Female	256 (63.7)
Male	146 (36.3)
Age (±S.D.)	57.85 (±15.83)
0–39	60 (14.9)
40–59	131 (32.6)
60–	211 (52.5)

**Table 2:** Summary of treatment success rates.

Protocol	Nr. of Total Success/Total Cases (%)	Nr. of Success/Total (%) 7 Days	Nr. of Success/Total (%) 10 Days	Nr. of Success/Total (%) 14 Days
AMO + CLA + PPI	127/171 (74.3)	65/93 (69.9)	57/72 (79.2)	5/6 (83.3)
AMO + CLA + PPI + BI	5/7 (71.4)	0/0 (0)	2/3 (66.67)	3/4 (75.0)
AMO + CLA + PPI + PRO	89/113 (78.8)	46/61 (75.4)	25/32 (78.1)	18/20 (90.0)
AMO + CLA + PPI + BI + PRO	31/37 (83.8)	0/1 (0)	12/16 (75.0)	19/20 (95.0)
TET + MET + PPI + BI	26/38 (68.4)	17/24 (70.8)	9/11 (81.8)	0/3 (0.0)
TET + MET + PPI + BI + PRO	33/36 (91.7)	0/0 (0)	8/11 (72.7)	25/25 (100.0)
**Total**	**311/402 (77.4)**	128/179 (71.5) *	113/145 (77.9) *	70/78 (89.7) *

* *p* = 0.0056 for comparison of total success rate for 7-, 10- and 14-day regimens.

**Table 3:** Comparison of AMO- and TET-based treatment protocols.

Protocol	Nr. of Total Success/Total Cases (%)	Nr. of Success/Total (%) 7 Days	Nr. of Success/Total (%) 10 Days	Nr. of Success/Total (%) 14 Days
Total AMO	252/328 (76.8) *	111/155 (71.6)	96/123 (78.0)	45/50 (90.0)
Total TET	59/74 (79.7) *	17/24 (70.8)	17/22 (77.3)	25/28 (89.2)

AMO-based protocols: AMO + CLA + PPI, AMO + CLA + PPI + BI, AMO + CLA + PPI + BI + PRO, TET-based protocols: TET + MET + PPI + BI TET + MET + PPI + BI + PRO. * *p* = 0.5902 for comparison of total success rate.

**Table 4:** Comparison of probiotic-containing and non-probiotic-containing treatment protocols.

Protocol	Nr. of Total Success/Total Cases (%)	Nr. of Success/Total (%) 7 Days	Nr. of Success/Total (%) 10 Days	Nr. of Success/Total (%) 14 Days
Total PRO	153/186 (82.3) *	46/62 (74.2)	45/59 (76.27)	62/65 (95.4) ▪
Total non-PRO	158/216 (73.1) *	82/117 (70.1)	68/86 (79.0)	8/13 (61.5) ▪

Total PRO: AMO + CLA + PPI + PRO, AMO + CLA + PPI + BI + PRO, Total non-PRO: AMO + CLA + PPI, AMO + CLA + PPI + BI, TET + MET + PPI + BI. * *p* = 0.0295 for comparison of total success rate; ▪ *p* = 0.0002 for comparison of success rate of 14-day regimens.

**Table 5:** Addition of probiotics to AMO-based protocols. Comparison of success rate of AMO + CLA + PPI and AMO + CLA + PPI + PRO. Comparison of success rate AMO + CLA + PPI and AMO + CLA + PPI + BI + PRO.

Protocol	Nr. of Total Success/Total Cases (%)	Nr. of Success/Total (%) 7 Days	Nr. of Success/Total (%) 10 Days	Nr. of Success/Total (%) 14 Days
AMO + CLA + PPI	127/171 (74.3)	65/93 (69.9) *	57/72 (79.2) **	5/6 (83.3) ***
AMO + CLA + PPI + PRO	89/113 (78.8)	46/61 (75.4) *	25/32 (78.1) **	18/20 (90.0) ***
AMO + CLA + PPI + BI + PRO	31/37 (83.8)	0/1 (0)	12/16 (75.0) ▪▪	19/20 (95.0) ▪▪▪

* *p* = 0.4554 in 7-day regimens; ** *p* = 0.9044 in 10-day regimens; *** *p* = 0.6539 in 14-day regimens; ▪▪ *p* = 0.7141 in 10-day regimens; ▪▪▪ *p* = 0.3469 in 14-day regimens.

**Table 6:** Probiotic supplementation to TET protocols.

Protocol	Nr. of Total Success/Total Cases (%)	Nr. of Success/Total (%) 7 Days	Nr. of Success/Total (%) 10 Days	Nr. of Success/Total (%) 14 Days
TET + MET + PPI + BI	26/38 (68.4)	17/24 (70.8)	9/11 (81.8)	0/3 (0.0) *
TET + MET + PPI + BI + PRO	33/36 (91.7)	0/0 (0)	8/11 (72.7)	25/25 (100.0) *

* *p* < 0.0001 for comparison of success rate in 14-day regimens.

**Table 7:** The effect of bismuth supplementation on treatment outcome.

Protocol	Nr. of Total Success/Total Cases (%)	Nr. of Success/Total (%) 7 Days	Nr. of Success/Total (%) 10 Days	Nr. of Success/Total (%) 14 Days
AMO + CLA + PPI	127/171 (74.3)	65/93 (69.9)	57/72 (79.2)	5/6 (83.3)
Total BI	31/45 (68.9)	17/24 (70.8)	11/14 (78.6)	3/7 (42.9)

Total BI: AMO + CLA + PPI + BI and TET + MET + PPI + BI.

**Table 8:** Effect of probiotics on bismuth-containing regimens.

Protocol	Nr. of Total Success/Total Cases (%)	Nr. of Success/Total (%) 7 Days	Nr. of Success/Total (%) 10 Days	Nr. of Success/Total (%) 14 Days
Total BI + PRO	64/73 (87.6) *	0/1 (0.0)	20/27 (74.1)	44/45 (97.8) ▪
Total BI	31/45 (68.9) *	17/24 (70.8)	11/14 (78.6)	3/7 (42.9) ▪

Total BI: AMO + CLA + PPI + BI and TET + MET + PPI + BI. Total BI + PRO: AMO + CLA + PPI + BI + PRO and TET + MET + PPI + BI + PRO; * *p* = 0.0124 for total success rate; ▪ *p* < 0.0001 in 14-day regimens.

**Table 9:** Comparison of bismuth containing agents.

Protocol	Nr. of Total Success/Total Cases (%)
Bismuth oxide (commercial)	30/35 (85.7) *
Bismuth citrate (magistral)	37/52 (71.2) *
Bismuth–metronidazol combination (magistral)	28/31 (90.3) *

* *p* = 0.0670.

**Table 10:** Investigated probiotic strains (serial Nr. for blinding commercial products was given in the time of occurrence order in the database).

Probiotic Product	Nr. of Strains	Strains
**Probiotic 1**	5	*Lactobacillus acidophilus*, *Lactobacillus bulgaricus*, *Bacillus coagulans*, *Bifidobacterium animalis lactis*, *Streptococcus thermophilus*
**Probiotic 2**	1	*Enterococcus faecium L3*
**Probiotic 3**	3	*Lactobacillus acidophilus* (*LA-5*), *Bifidobacterium* (*BB-12*), *Lactobacillus paracasei* (*L. CASEI 431*)
**Probiotic 4**	7	* Lactobacillus casei*, *Lactobacillus rhamnosus*, *Streptobacillus thermophilus*, *Lactobacillus acidophilus*, *Bifidobacterium breve*, *Bifidobacterium longum*, *Lactobacillus bulgaricus*
**Probiotic 5**	1	* Saccharomyces boulardii CNCM I-745 *
**Probiotic 6**	9	*Bifidobacterium bifidum W23*, *Bifidobacterium lactis W51*, *Bifidobacterium lactis W52*, *Lactobacillus acidophilus W22*, *Lactobacillus casei W56*, *Lactobacillus paracasei W20*, *Lactobacillus plantarum W62*, *Lactobacillus salivarius W24*, *Lactococcus lactis W19*
**Probiotic 7**	1	*Lactobacillus rhamnosus GG/Lactobacillus acidophilus LA-5*
**Probiotic 8**	2	* Lactobacillus reuteri ATCC PTA 6475*, *Lactobacillus reuteri Protectis^®^ DSM 17938*
**Probiotic 9**	1	* Bacillus clausii *
**Probiotic 10**	14	*Lactobacillus paracasei PXN^®^ 37™*, *Lactobacillus plantarum PXN^®^ 47™*, *Lactobacillus rhamnosus PXN^®^ 54™*, *Bacillus subtilis PXN^®^ 21^®^*, *Bifidobacterium bifidum PXN^®^ 23™*, *Bifidobacterium breve PXN^®^ 25™*, *Bifidobacterium longum PXN^®^ 30™*, *Lactobacillus helveticus PXN^®^ 35™*, *Lactococcus lactis ssp. lactis PXN^®^ 63™*, *Streptococcus thermophilus PXN^®^ 66™*, *Bifidobacterium infantis PXN^®^ 27™*, *Lactobacillus delbrueckii* ssp. *bulgaricus PXN^®^ 39™*, *Lactobacillus helveticus PXN^®^ 45™*, *Lactobacillus salvarius PXN^®^ 57™.*

**Table 11:** Comparison of the efficacy of probiotic-containing multiple vs. single strains.

Probiotics	Serial Nr.	Nr. of Success/Total (%)
***Multiple strain***	Probiotic 1	7/11 (63.6)
	Probiotic 3	15/22 (68.2)
	Probiotic 4	2/4 (50)
	Probiotic 6	10/10 (100.0)
	Probiotic 8	53/57 (93.0)
	Probiotic 10	1/1 (100.0)
	**Total**	88/105 (83.8) *
**Probiotics**	**Serial Nr.**	**Nr. of Success/Total (%)**
***Single strain***	Probiotic 2	20/27 (74.1)
	Probiotic 5	23/30 (76.7)
	Probiotic 7	3/4 (75.0)
	Probiotic 9	1/1 (100.0)
	**Total**	47/62 (75.6) *

* *p* = 0.2042 for comparison of success rate for multiple vs. single strains.

**Table 12:** The additional value of probiotic choice.

Protocol	Probiotic Not Specified	Probiotic 1	Probiotic 2	Probiotic 3
AMO + CLA + PPI + PRO	17/18 (94.4)	7/11 (63.6) *	20/26 (76.9) *	14/19 (73.7) *
AMO + CLA + PPI + BI + PRO	0/0 (0.0)	0/0 (0.0)	0/1 (0.0)	0/0 (0.0)
TET + MET + PPI + BI + PRO	1/1 (100.0)	0/0 (0.0)	0/0 (0.0)	1/3 (33.33)
**Total**	**18/19 (94.7)**	**7/11 (63.6)**	**20/27 (74.1)**	**15/22 (68.2)**
**Protocol**	**Probiotic 4**	**Probiotic 5**	**Probiotic 6**	**Probiotic 7**
AMO + CLA + PPI + PRO	0/2 (0.0)	20/26 (76.9) *	3/3 (100.0)	3/3 (100.0)
AMO + CLA + PPI + BI + PRO	0/0 (0.0)	2/2 (100.0)	6/6 (100.0) ▪	0/1 (0.0)
TET + MET + PPI + BI + PRO	2/2 (100.0)	1/2 (50.0)	1/1 (100.0)	0/0 (0.0)
**Total**	**2/4 (50.0)**	**23/30 (76.7)**	**10/10 (100.0)**	**3/4 (75.0)**
**Protocol**	**Probiotic 8**	**Probiotic 9**	**Probiotic 10**	
AMO + CLA + PPI + PRO	3/3 (100.0)	1/1 (100.0)	1/1 (100.0)	
AMO + CLA + PPI + BI + PRO	23/27 (85.2) ▪	0/0 (0.0)	0/0 (0.0)	
TET + MET + PPI + BI + PRO	27/27 (100.0)	0/0 (0.0)	0/0 (0.0)	
**Total**	**53/57 (93.0)**	**1/1 (100.0)**	**1/1 (100.0)**	

* *p* = 0.8380 for comparison of success rate for Probiotic 1, Probiotic 2, Probiotic 3 and Probiotic 5 preparations for AMO + CLA + PPI + PRO protocols; *▪ p* = 0.3146 for comparison of success rate for Probiotic 6 and Probiotic 8 preparations for AMO + CLA + PPI + BI + PRO protocols.

**Table 13:** Summary of results.

Goal	Result
*Determination of optimal treatment duration*	14-day regimens are significantly better than 7- and 10-day regimens in eradication success
*Finding the protocols with the highest eradication rates*	AMO + CLA + PPI + PRO, AMO + CLA + PPI + BI + PRO, TET + MET + PPI + BI + PRO are the most effective protocols amongst 14-day treatments, with comparable success rates between the different regimes
*To clarify whether addition of probiotics improves outcomes in different settings*	Probiotic-containing protocols are more successful compared to non-probiotic-containing regimens, and their effect is more prominent when bismuth is also administered
*To clarify the effect of bismuth addition to clarithromycin-based protocols*	Bismuth addition itself has no effect on the success of clarithromycin-based treatments (AMO + CLA + PPI + BI vs. AMO + CLA + PPI)
*To investigate if any of the bismuth compounds are superior in eradication success*	No significant effect was observed in success rates amongst the different bismuth types (commercial bismuth oxide, magistral bismuth citrate, magistral bismuth–metronidazole)
*To find the most effective probiotic strain*	Based on treatment success, the most effective probiotic strain was the combination of *Lactobacillus reuteri ATCC PTA 6475* and *Lactobacillus reuteri Protectis^®^ DSM 17938* with a success rate of 85.2% when added to AMO + CLA + PPI + BI + PRO, and 100% in combination with TET + MET + PPI + BI + PRO as the “PRO” part.
*To give recommendation for H. pylori eradication in a clarithromycin-resistant and tetracycline/bismuth naïve area*	We suggest the use of a tetracycline-based bismuth and probiotic-supplemented regimen (TET + MET + PPI + BI + PRO) as first line treatment to maximize treatment success. Furthermore, we suggest the use of a 14-day bismuth and probiotic-supplemented, clarithromycin-based protocol (AMO + CLA + PPI + BI + PRO) to achieve comparable outcomes to the TET + MET + PPI + BI + PRO protocol, when tetracycline-based regimens cannot be used.

**Table 14:** Treatment combinations: standard regimens and their supplementations. “X” marks explain the exact composition of the protocols, whether they are bismuth and/or probiotics supplemented.

Standard	Bismuth	Probiotics
AMO + CLA + PPI		
AMO + CLA + PPI + BI	x	
AMO + CLA + PPI + PRO		x
AMO + CLA + PPI + BI + PRO	x	x
TET + MET + PPI + BI	x	
TET + MET + PPI + BI + PRO	x	x

**Table 15:** Treatment combinations, including dosage.

Protocol	Used Agents	Dose
**AMO + CLA + PPI**	Amoxicillin	2 × 1000 mg
	Clarithromycin	2 × 500 mg
	PPI	standard dose
**AMO + CLA + PPI + BIZ**	Amoxicillin	2 × 1000 mg
	Clarithromycin	2 × 500 mg
	PPI	standard dose
	Bismuth oxide/bismuth citrate	see Table 16.
**AMO + CLA + PPI + PRO**	Amoxicillin	2 × 1000 mg
	Clarithromycin	2 × 500 mg
	PPI	standard dose
	Probiotics	any strain
**AMO + CLAV+ PPI + BIZ + PRO**	Amoxicillin	2 × 1000 mg
	Clarithromycin	2 × 500 mg
	PPI	standard dose
	Bismuth citrate/Bismuth oxide	see Table 16.
	Probiotics	any strain
**TET + MET +PPI + BIZ**	Tetracycline	4 × 500 mg
	Metronidazol	2 × 500 mg
	PPI	standard dose
	Bismuth oxide/bismuth citrate/bismuth metronidazole	see Table 16. (in cases when bismuth-metronidazole was given this combination substitutes both bismuth and metronidazole)
**TET + MET + BIZ + PRO**	Tetracycline	4 × 500 mg
	Metronidazol	2 × 500 mg
	PPI	standard dose
	Bismuth	see Table 16. (in cases when bismuth-metronidazole was given this combination substitutes both bismuth and metronidazole)
	Probiotics	any strain

**Table 16:** Investigated bismuth compounds.

Drug or Formulation	Used Dose
Bismuth-oxide (commercial drug)	4 × 120 mg
Bismuth citrate (magistral)	4 × 120 mg
Bismuth-metronidazol combination (magistral)	Bismuth citrate: 4 × 120 mg, metronidazole: 4 × 375 mg (one capsule contains 120 mg bismuth citrate and 375 mg metronidazole)

## Data Availability

For ethical and confidentiality reasons, the data presented in this study are available upon request from the corresponding author.

## Abbreviations

The following abbreviations are used in this manuscript. Abbreviations that were used in the probiotic strain description are a copy from summary of product characteristics of each compound.

*H. pylori*	Helicobacter pylori
SAT	stool *H. pylori* antibody test
AMO	amoxicillin
CLA	clarithromycin
PPI	proton pump inhibitor
BI	bismuth
TET	tetracycline
MET	metronidazole
RCT	randomized controlled trial
AMO protocols	amoxicillin–clarithromycin-based regimens, traditionally these protocols called as “clarithromycin-based” in scientific literature
TET protocols	tetracycline-metronidazole based protocols
